# *Ba813* harboring *Bacillus cereus*, genetically closely related to *Bacillus anthracis*, causing nosocomial bloodstream infection: Bacterial virulence factors and clinical outcome

**DOI:** 10.1371/journal.pone.0235771

**Published:** 2020-07-13

**Authors:** Tetsuji Aoyagi, Kengo Oshima, Shiro Endo, Hiroaki Baba, Hajime Kanamori, Makiko Yoshida, Koichi Tokuda, Mitsuo Kaku

**Affiliations:** 1 Department of Infectious Diseases, Internal Medicine, Tohoku University Graduate School of Medicine, Sendai, Japan; 2 Department of Infection Control and Laboratory Diagnostics, Internal Medicine, Tohoku University Graduate School of Medicine, Sendai, Japan; 3 Department of Infectious Diseases, Tohoku Medical and Pharmaceutical University, Sendai, Japan; Defense Threat Reduction Agency, UNITED STATES

## Abstract

*Bacillus cereus* commonly causes catheter-related bloodstream infections (BSIs) in hospital settings, and occasionally occurs fatal central nervous system (CNS) complications. *B*. *cereus* harboring *Ba813*, a specific chromosomal marker of *Bacillus anthracis*, has been found in patients with severe infection and nosocomial BSI. However, the bacteriological profile and clinical feature of *Ba813* (+) *B*. *cereus* are unclear. Fifty-three patients with *B*. *cereus* BSI were examined. Isolates were evaluated for *Ba813*, *B*. *anthracis*-related and food poisoning-related virulence, multilocus sequencing typing, and biofilm formation. Patients’ clinical records were reviewed retrospectively. The 53 isolates were comprised of 29 different sequence types in two distinct clades. Seventeen of the 53 (32%) *B*. *cereus* isolates including five sequence types possessed *Ba813* and were classified into Clade-1/Cereus-III lineage which is most closely related to Anthracis lineage. No *B*. *cereus* possessed *B*. *anthracis*-related virulence genes. *Ba813* (+) strains showed a lower prevalence of enterotoxin genes than Clade-2 strains (n = 4), but no difference from Clade-1. *Ba813* (+) strains showed significantly lower biofilm formation than Clade-1/non-Cereus-III (n = 22) and Clade-2 strains, respectively. Compared to Clade-1/non-Cereus-III and Clade-2 *B*. *cereus*, *Ba813* (+) strains were isolated more frequently from elderly patients, patients with indwelling central venous catheter rather than peripheral venous catheter, and patients who remained in the hospital for longer before BSI onset. No significant differences in disease severity or mortality were observed. Though two of the ten *Ba813* (-) strains in Clade-1/Cereus III were isolated from the patients with CNS complication, no significant difference was observed in the bacterial profile and clinical characteristics among Clade-1/Cereus III strains. In conclusion, our report suggested that *Ba813-*harboring *B*. *cereus* strains, genetically closely related to *B*. *anthracis*, were abundant among *B*. *cereus* strains in the hospital setting, and might cause catheter-related nosocomial BSI. However, it did not affect the clinical outcomes.

## Introduction

*Bacillus cereus*, a gram-positive bacillus, is widely distributed in natural environments including water and soil. *B*. *cereus* is also an important pathogen causing food poisoning and can cause fatal systemic infection, particularly in immunocompromised patients [1]. The most common clinical feature of *B*. *cereus* infection in hospitalized patients is catheter-related bacteremia [2]. Among immunosuppressed patients, *B*. *cereus* nosocomial blood stream infection (BSI) can have fatal outcomes complicated with central nerve systemic (CNS) infection, meningitis, and brain abscess [1, 2].

Food poisoning due to *B*. *cereus* results in diarrhea and vomiting, and is closely associated with emetic toxin and enterotoxin [3, 4]. However, the pathogenicity of *B*. *cereus* that causes systemic infections including nosocomial BSI remains unclear. In previous reports, *B*. *cereus* strains G9241 [5] and 03BB102 [6], which are genetically close to *B*. *anthracis*, produce B. anthracis toxin and/or capsular toxins and cause community-onset pneumonia and fatal outcomes in healthy persons. *Ba813*, a 277-base pair fragment encoded on *B*. *anthracis* chromosomal DNA, has been used to differentiate *B*. *anthracis* and *B*. *cereus* [7]. However, some *B*. *cereus* strains possess *Ba813* [8, 9]. *Ba813-*harboring *B*. *cereus* has been isolated from a patient with septic shock arising due to wound infection after surgery [10]. Moreover, recent studies have shown that this bacteria might be associated with outbreaks of nosocomial BSI [11, 12]. Some *Ba813*-harboring *B*. *cereus* possess capsular toxin genes found in *B*. *anthracis* [11]. Though *Ba813* is considered to a crucial genetic marker in *B*. *cereus* isolated from hospitalized patients, the distribution and clinical characteristics of *Ba813*-harboring *B*. *cereus* BSI in the hospital setting is unknown.

Multiloucus sequence typing (MLST) studies detect genetic variation and have been widely used examine the phylogeny of *B*. *cereus* group species including *B*. *anthracis* [12–14]. The *B*. *cereus* group is divided into three clades (Clade-1–3), with clinical isolates of *B*. *cereus* classified mainly in Clade-1 and -2 [14]. The Clade-1 consists of four major lineages: Cereus-I–III and Anthracis [13]. The Clade-1/Cereus-III lineage is most closely related to the Anthracis lineage; 03BB102 *B*. *cereus* and some *Ba813* (+) *B*. *cereus* were previously shown to include the Clade 1/Cereus III lineage [12, 14].

In this study, we analyzed the molecular and biological differences *Ba813*-harboring strains among *B*. *cereus* clinical isolates and assessed the impact of *Ba813*-harboring *B*. *cereus* on the clinical characteristics and outcomes of *B*. *cereus* nosocomial BSI.

## Materials and methods

### Patients and study design

In total, 53 patients (≥18 years old) with *B*. *cereus* BSI were enrolled retrospectively from January 2009 to December 2012 at Tohoku University Hospital, a 1200-bed tertiary-care teaching hospital in Japan. BSI was based on more than two positive blood culture bottles with ≥2 criteria for systemic inflammatory response syndrome (SIRS) [15]. The following data were collected from each patient’s electronic medical record: age, sex, co-morbidities, site of hospitalization, and risk factors for *B*. *cereus* BSI; insertion of vascular access catheter, recent surgery, treatment with immunosuppressive drugs, and administration of amino acid preparation and antimicrobial agents [1, 16–18]. Overall co-morbidities were assessed using the Charlson comorbidity index. An amino acid preparation was defined as an infusion preparation containing >15 g amino acids per 500 mL. The classification criteria of sepsis and related conditions (SIRS, severe sepsis, and septic shock) were used to evaluate diseases severity [15]. Complication by central nervous system (CNS) lesions were evaluated by magnetic resonance imaging. Persistent bacteremia was defined as bacteremia persisting for ≥7 days after initiating appropriate therapy. The outcomes studied included 30-day mortality, all-cause mortality, and length of hospital stay. Clinical isolates were collected as part of routine microbiology laboratory tests. No written informed consent was required because it was a retrospective observational study. This study was approved by the Ethics Committees of Tohoku University (2014-1-292), and ethics committee waived the requirement for informed consent. All patient data were fully anonymized before access for analysis.

### Microbiological molecular analysis

For MLST, seven housekeeping genes (*glp*, *gmk*, *ilvD*, *pta*, *pur*, *pcyA*, *tpi*) and PCR conditions was referenced from the *B*. *cereus* MLST website (http://www.pubmlst.org/bcereus). Purified PCR products were sequenced using a BigDyeTM Terminator v3.1 cycle sequencing kit according to the manufacturer’s instructions on an Applied Biosystems 3130xl Genetic Analyzer (Foster City, CA, USA). To analyze the relationship of the 53 strains with other strains such as *B*. *anthracis*, highly pathogenic *B*. *cereus* (03BB102, 03BB018, G9241) and *B*. *cereus* from ATCC (Manassas, VA, USA) strains and clinical isolates in Japan collected from the *B*. *cereus* MLST database (https://pubmlst.org/bcereus/) and previous studies [12, 19], a neighbor-joining tree (p-distance) with 1,000 bootstrap replications was constructed in MEGA X program [20]. The presence or absence of the following virulence genes by polymerase chain reaction (PCR): *Ba813* (a 277-base pair chromosomal DNA fragment from *B*. *anthracis*), and *B*. *anthracis*-related and foodborne virulence genes were examined via PCR (S1 Table).

### Biofilm assay in microtiter plate

*B*. *cereus* isolates were adjusted to 5 × 10^5^ cfu/mL in LB broth, and added to 200 μL of LB broth in a 96-well polystyrene plate. After incubation for 48 h at 35°C, the cells attached to the plate were stained with 0.1% crystal violet and absorbance was measured at 595 nm [21].

### Statistical analysis

Descriptive statics, such means, standard deviations, frequencies, and percentages, were collected. Statistical analyses were performed using GraphPad Prism 8 software (GraphPad, Inc., La Jolla, CA, USA). Differences between patient data were analyzed using the chi-squared test or Fisher exact test as appropriate. Mean values were compared by one-way analysis of variance, followed by the Tukey multiple-comparison test, for comparisons between more than two groups. *p* < 0.05 was considered as statistically significant.

## Results

### Phylogenetic analysis of *Ba813* (+) *B*. *cereus* isolated from blood cultures

**Fig 1:**
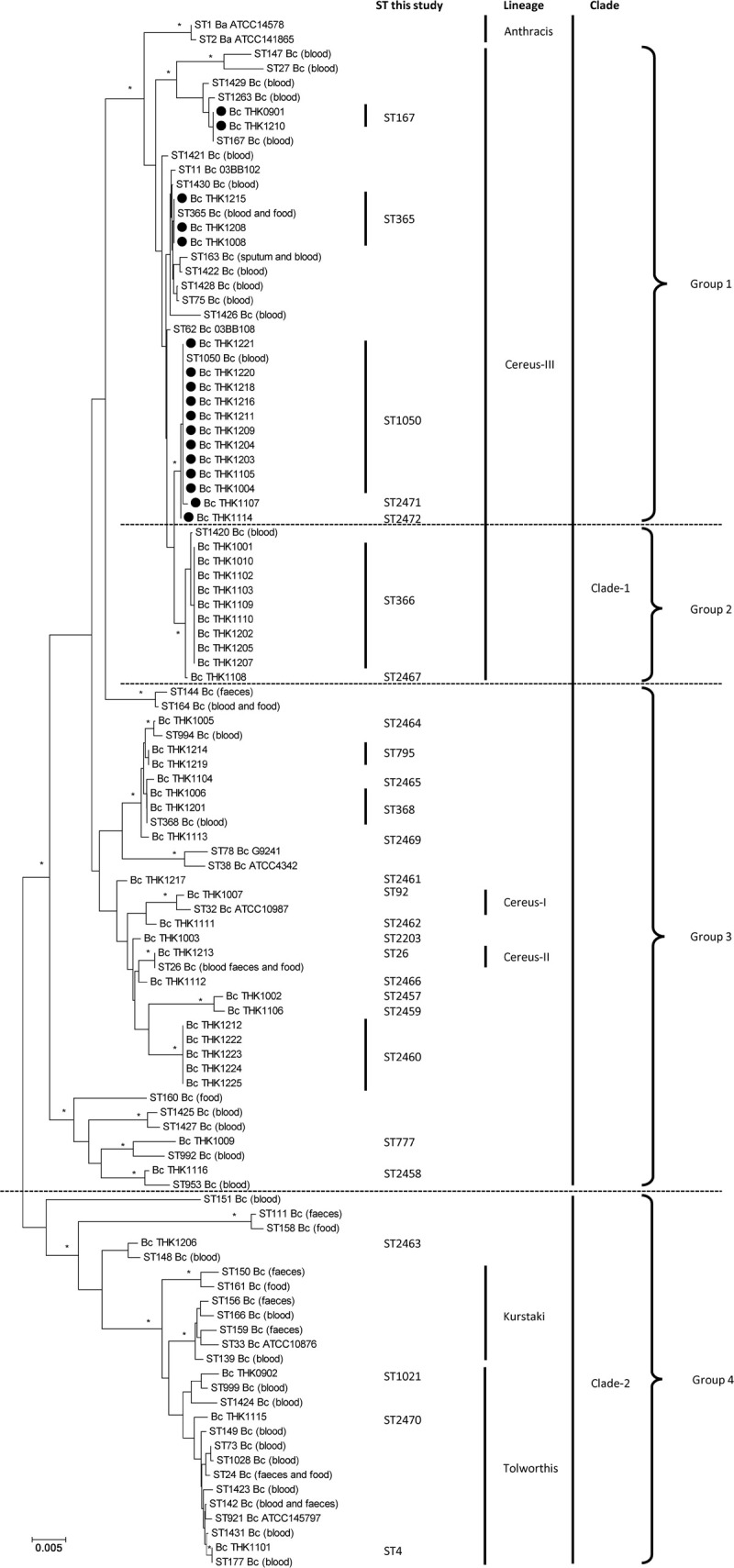
Phylogenetic analysis based on MLST of *B*. *cereus and B*. *anthracis*. Differences in MLST among *B*. *cereus* of 53 clinical isolates and selected strains of *B*. *anthracis* and *B*. *cereus* collected from the MLST database (http://www.pubmlst.org/bcereus) and previous reports in Japan were compared. We collected *B*. *anthracis*, high pathogenic *B*. *cereus* (03BB102, 03BB108, and G9241), and *B*. *cereus* which were ATCC strains and other clinical isolates in Japan. A phylogenetic tree was drawn by MEGA X using neighbor-joining methods [20]. All nodes were supported by 1,000 bootstrap replications. Clade and lineage were defined as described previously [13]. Scale bar, 0.005 substitutions per nucleotide. Asterisk represents branches with ≥95% bootstrap support. Closed circle: *Ba813*(+) *B*. *cereus*; Ba: *B*. *anthracis*; Bc: *B*. *cereus*; ST; sequence type.

Of the 53 patients with *B*. *cereus* bacteremia, 17 (32.1%) *B*. *cereus* isolates possessed *Ba813*. The phylogenetic relationships of *Ba813* (+) *B*. *cereus* with other *B*. *cereus* stains were analyzed by MLST (Fig 1). Among the 53 strains, 29 different MLST genotypes were identified, 16 of which were new to the MLST database. Forty-nine *B*. *cereus* isolates were classified into the *Bacillus* group Clade-1. Twenty-seven strains (51%) were grouped into the Cereus-III lineage which were closely related to *B*. *anthracis* and *B*. *cereus*

03BB102 and 03BB108. The *Ba813* (+) *B*. *cereus* strains were divided into following five different sequencing types (ST): ST365, ST167, ST1050, and two new ST (ST2471 and ST2472), which had the same allele numbers at six of seven loci with ST1050. Interestingly, two STs including ST366 and new ST2467 which had the same allele numbers at six of seven with ST366 were found in Cereus-III, but these strains did not possess *Ba813*. In Cereus-III linage, *Ba813* (-) *B*. *cereus* was statistically separated from *Ba813* (+) *B*. *cereus*. Thus, to understand the impact of *Ba813* (+) *B*. *cereus* on the bacteriological and clinical characteristics of BSI compared to other *B*. *cereus* strains, we divided the 53 isolates into four groups, Group 1: *Ba813* (+) *B*. *cereus* in Clade-1/Cereus-III; Group 2: *Ba813* (-) *B*. *cereus* in Clade-1/Cereus-III; Group 3: *Ba813* (-) *B*. *cereus* in Clade-1/non-Cereus-III; Group 4: *B*. *cereus* in Clade-2.

### Detection of genes encoding virulence factors in *Ba813* (+) *B*. *cereus*

Table 1 shows the prevalence of virulence genes associated with *B*. *anthracis* and *B*. *cereus*-food poising. None of the 53 isolates carried genes associated with toxins (*pag*, *cya*, and *lef*) or capsular (*cap*) synthesis. Whereas all *B*. *cereus* strains except for two strains harbored *nheA*, *nheB*, *and nheC*, only eight strains harbored *bhl* genes. None of the *B*. *cereus* isolates carried *cytk*. Strains possessing *bhlA*, *bhlC* and/or *bhlD* produced enterotoxin according to reversed passive latex agglutination assay. All strains classified into Clade-2 (Group 4) had *bhl* genes and produced enterotoxin. The prevalence of virulence genes among Clade-1 strains did not significantly differ.

**Table 1:** Genes encoding pathogenic factors in *B*. *cereus* isolated from the patients with BSI.

	Clade	Cereus lineage	Number	*hblA*	*hblC*	*hblD*	*nheA*	*nheB*	*nheC*	*cytk*	*pag*	*cya*	*lef*	*cap*
Group 1	1	III	17	2	2	1	16	16	17	0	0	0	0	0
(*Ba813* positive)				(11.8%)	(11.8%)	(5.9%)	(94.1%)	(94.1%)	(100%)	(0%)	(0%)	(0%)	(0%)	(0%)
Group 2	1	III	10	1	1	1	10	10	10	0	0	0	0	0
(*Ba813* negative)				(10.0%)	(10.0%)	(10.0%)	(100%)	(100%)	(100%)	(0%)	(0%)	(0%)	(0%)	(0%)
Group 3	1	―	22	0	0	0	22	21	22	0	0	0	0	0
(*Ba813* negative)				(0%)	(0%)	(0%)	(100%)	(95.5%)	(100%)	(0%)	(0%)	(0%)	(0%)	(0%)
Group 4	2	―	4	4	4	3	4	4	4	0	0	0	0	0
(*Ba813* positive)				(100%)	(100%)	(100%)	(100%)	(100%)	(100%)	(0%)	(0%)	(0%)	(0%)	(0%)

### Biofilm activity in *Ba813* (+) *B*. *cereus*

**Fig 2:**
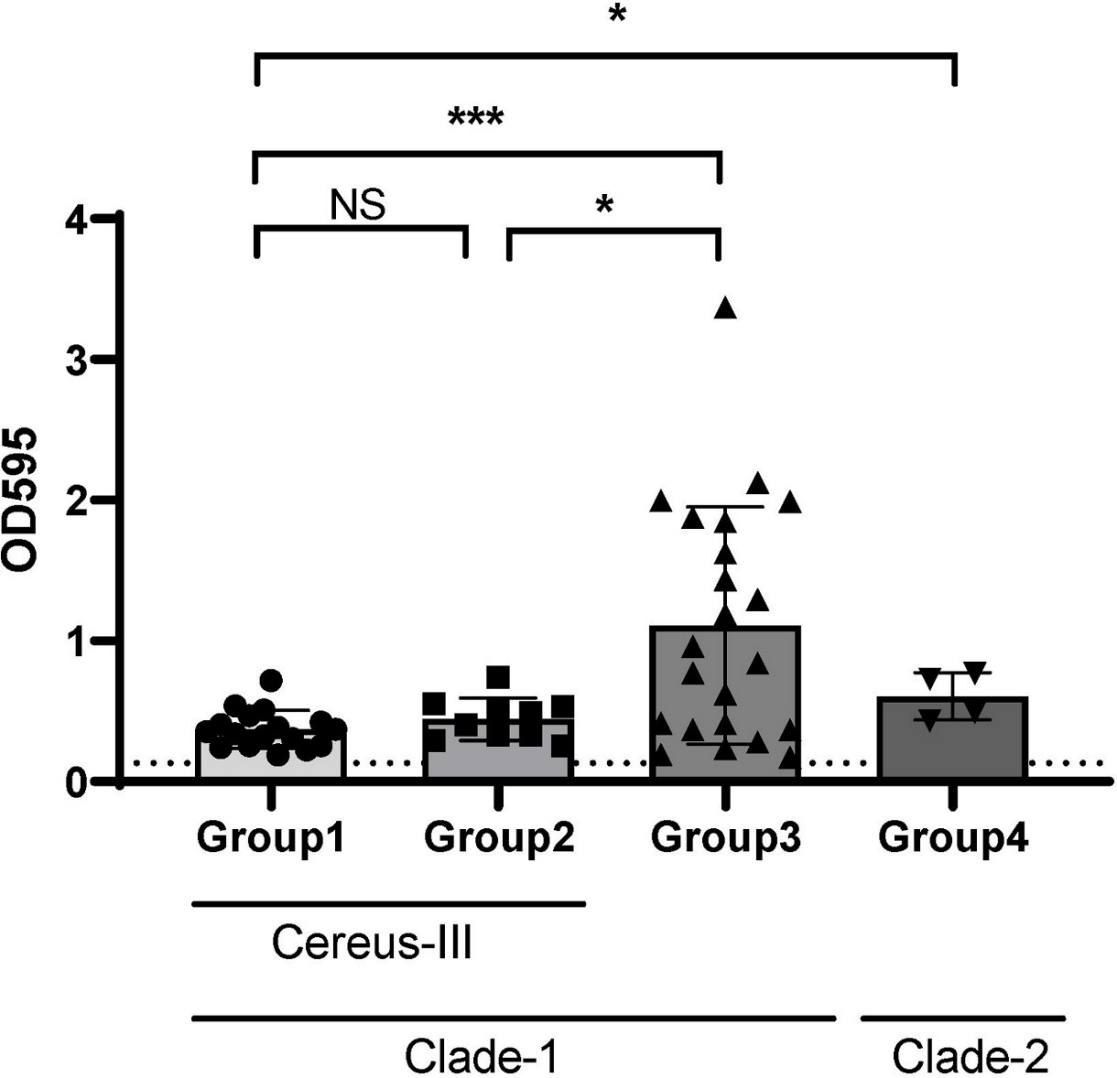
Difference of biofilm formation among the three groups. Biofilm formation was determined by measuring the OD595 nm after crystal violet staining. An absorbance value <0.13 represents a biofilm-negative strain. Three independent biological experiments were performed, each with four technical replicates. The data are presented as the mean ± SD. Cut-off value was defined as two standard deviations above the mean of negative control. NS: not significant, *p < 0.05, ***p < 0.001.

*B*. *cereus* is known to produce biofilms which enhances its attachment to the catheter [21, 22]. We confirmed that all *B*. *cereus* strains formed biofilm in a microplate assay system (Fig 2). The level of biofilm formation in *Ba813* (+) *B*. *cereus* (Group 1) was significantly lower than that in *B*. *cereus* in Clade-1/non-Cereus-III (Group 3) and *B*. *cereus* in Clade-2 (Group4). However, no difference in biofilm formation was observed between Groups 1 and 2 *B*. *cereus*.

### Clinical characteristics and outcomes of bacteremia caused by *Ba813* (+) *B*. *cereus*

The clinical characteristics of the 53 patients are shown in Table 2. Patients with Group 1 *B*. *cereus* were significantly older than patients with Groups 3 and 4 *B*. *cereus*. However, no difference in age was found in Groups 1 and 2 *B*. *cereus*. All cases showed onset in the hospital, with bacteria isolated more than 48 h after admission. The length of hospital stay before the onset of BSI with Group 1 *B*. *cereus* was significantly longer than those with *B*. *cereus* in Groups 3 and 4, but no differences were observed between Groups 1 and 2. There was no difference in underlying diseases, incidence in inpatient setting, and prior medication among patients infected with the four different *B*. *cereus* groups.

**Table 2:** Comparison of clinical characteristics and outcome among four groups of *B*. *cereus* BSI.

	Group 1		Group2		Group3		Group 4		*p*-value
	Ba813 (+) Clade-1/Cereus-III		Ba813 (-) Clade-1/Cereus-III		Ba813 (-) Clade-1/non-Cereus-III		Ba813 (-) Clade-2		
	(n = 17)		(n = 10)		(n = 22)		(n = 4)		
age (median, 25–75%)	65	(51–73)	66	(53–79)	50	(34–67)	36	(27–47)	<0.05 a, b, c, d
Male, No (%)	9	(52.9%)	2	(20.0%)	11	(50.0%)	2	(50.0%)	NS
Days of BSI onset after admission (median, 25–75%)	34	(12–139)	54	(21–110)	24	(12–45)	22	(12–46)	<0.05 a, b, c, d
**Underlying diseases, No (%)**									
Cardiovascular disease	6	(35.3%)	3	(30.0%)	3	(13.6%)	0	(0.0%)	NS
Hematological malignancy	2	(11.8%)	3	(30.0%)	1	(4.5%)	0	(0.0%)	
Solid malignancy	5	(29.4%)	1	(10.0%)	7	(31.8%)	0	(0.0%)	
Diabetes mellitus	5	(29.4%)	2	(20.0%)	1	(4.5%)	1	(25.0%)	
Chronic renal failure	2	(11.8%)	2	(20.0%)	1	(4.5%)	1	(25.0%)	
Autoimmune disease	1	(5.9%)	0	0	2	(9.0%)	0	(0.0%)	
Charlson co-morbidity index (mean, SD)	3.4 (±2.6)		2.8 (±2.5)		2.6 (±3.1)		1.5 (±1.3)		NS
Neutropenia (WBC<500 cell/m^3^)	1	(5.9%)	2	(20.0%)	1	(4.5%)	0	(0%)	NS
**Hospital setting, No (%)**									
Medical ward	8	(47.1%)	5	(50.0%)	8	(36.4%)	2	(50.0%)	NS
Surgical ward	4	(23.5%)	2	(20.0%)	6	(27.2%)	0	(0.0%)	
ICU (including emergency room)	5	(29.4%)	3	(30.0%)	8	(36.4%)	2	(50.0%)	
**Medical device, No (%)**									
Central venous catheter	7	(41.1%)	4	(40.0%)	1	(4.5%)	0	(0.0%)	<0.05 a, c
Peripheral venous catheter	10	(58.9%)	6	(60.0%)	21	(95.5%)	4	(100%)	
**Prior medication, No (%)**									
Immunosuppressive treatment^†^	4	(23.5%)	5	(50.0%)	6	(23.1%)	2	(50.0%)	NS
Intravenous aminoacid preparation (within 72h)	13	(76.5%)	10	(100%)	20	(90.9%)	2	(50.0%)	NS
Antimicrobial agents (within 72h)	3	(17.6%)	6	(60.0%)	10	(38.5%)	2	(50.0%)	NS
Surgical operation	4	(23.5%)	3	(30.0%)	9	(40.9%)	1	(25.0%)	NS
**Severity of illness, No (%)**									
Sepsis (SIRS)^‡^	15	(88.2%)	7	(70.0%)	19	(86.4%)	4	(100%)	NS
Sever sepsis^‡^	0	(0.0%)	0	(0.0%)	1	(4.5%)	0	(0.0%)	
Septic shock^‡^	2	(11.8%)	3	(30.0%)	2	(9.1%)	0	(0.0%)	
Complication with CNS^※^	0	(0.0%)	2	(20.0%)	0	(0.0%)	0	(0.0%)	NS
Persistent bacteremia^§^	3	(17.6%)	2	(20.0%)	2	(9.1%)	0	(0.0%)	NS
**Treatment, No (%)**									
Vancomycin or Teicoplanin	6	(35.3%)	4	(40.0%)	12	(54.5%)	1	(25.0%)	NS
Carbapenem	7	(41.2%)	3	(30.0%)	8	(36.4%)	0	(0.0%)	
Quinolone	3	(17.6%)	3	(30.0%)	4	(18.2%)	2	(50.0%)	
Other antimicrobials^¶^	2	(11.6%)	1	(10.0%)	1	(4.5%)	1	(25.0%)	
Combination therapy	2	(11.8%)	2	(20.0%)	3	(13.6%)	0	(0.0%)	NS
**Clinical outcome**									
30-days mortality, No (%)	1	(5.9%)	0	(0.0%)	1	(13.6%)	0	(0.0%)	NS
all-cause mortality, No (%)	2	(11.8%)	2	(20.0%)	3	(11.5%)	0	(0.0%)	NS
Duration of therapy (median, 25–75%)	8	(6.0–10)	12	(3.3–16)	10	(6.3–14)	9	(5.3–11.8)	NS
Median LOHS after bacteremia (median, 25–75%)	49	(20–94)	55	(22–97)	37	(22–106)	58	(28–106)	NS

BSI, bloodstream infection; ICU, intensive care unit; SIRS, systemic inflammatory response syndrome; CNS, central nerve system; LOHS, length of hospital stay; NS: not significantly

^†^ Immunosuppressive therapy was included in corticosteroids, anticancer agents and another immunosuppressant drug

^‡^ Sepsis (SIRS) is defined two or more systemic inflammatory response criteria. Severe sepsis is defined as sepsis with acute organ dysfunction, and septic shock is a form of severe sepsis where the organ dysfunction involves the cardiovascular system [15].

^※^Complication by central nervous system (CNS) lesions were evaluated by magnetic resonance imaging.

^§^Persistent bacteremia was defined as bacteremia persisting for ≥7 days after initiating appropriate therapy.

^¶^ Other antimicrobials of treatment for *B*. *cereus* were included in clindamycin and aminoglycosides.

a (Group 1 vs Group 3), b (Group 1 vs Group 4), c (Group 2 vs Group 3), d (Group vs Group 4).

All cases in this study had indwelling peripheral venous catheter (PVC) or central venous catheter (CVC). Among the patients with *B*. *cereus* in Clade-1, the proportions of indwelling PVC in patients with Groups 1 and 2 *B*. *cereus* were significantly lower than that in patients with Group 3 *B*. *cereus*.

Eight patients (15.0%) with *B*. *cereus* bacteremia had severe sepsis or septic shock; however, no difference in disease severity was observed between patients with Group 1 *B*. *cereus* and other groups of *B*. *cereus*. Seven patients (13.2%) had persistent bacteremia, but no difference was observed among the four groups. Two patients (3.8%) had CNS complications (intracranial hemorrhage), and *B*. *cereus* (ST-366) in Group 2 was isolated from these patients.

Two (3.8%) patients died within 30 days of onset of *B*. *cereu*s BSI, and all-cause mortality during hospital stays was 15.0%. However, there was no significant difference in the treatment characteristics and clinical outcomes between the four study groups.

## Discussion

Some *B*. *cereus* strains harboring *B*. *anthracis* virulent plasmids or genetically closely related to *B*. *anthracis* have been isolated from patients with severe infectious diseases in community settings [5, 6, 23]. In hospital settings, severe *B*. *cereus* infection such as pneumonia [24] and CNS infection [25, 26] have been reported. However, the impact of *B*. *cereus* strains, which have a similar genetic background as *B*. *anthracis*, on clinical features in nosocomial BSI is unknown.

Historically, *B*. *anthracis* harbors two plasmid, pXO1 and pXO2, carrying genes for toxin synthesis and capsule synthesis, respectively. However, *B*. *cereus* strains harboring pXO-1 and pXO2-like plasmids, named as *B*. *cereus* Biovar *anthracis*, were recently isolated as the causative pathogens of anthrax-like infections in primates in South Africa [27, 28]. Some *B*. *cereus* strains possessing the *B*. *anthracis* cap have been isolated from patients with severe community acquired pneumonia [23] as well as nosocomial BSI in Japan [11]. Although we did not detect *B*. *cereus* strains harboring *B*. *anthrax*-related virulence genes, 32% (17/53) of *B*. *cereus* strains harboring *Ba813* were isolated sporadically from patients with BSI in the hospital setting, and were genetically closely related to *B*. *anthracis* by MLST analysis. Additionally, two previous studies in Japan, which evaluated sequence typing of *B*. *cereus* isolated from blood culture obtained from five hospitals, showed that 32 (65%) of the 49 isolates were classified into Clade-1/Cereus-III, and more than 80% of the isolates in Clade-1/Cereus-III possessed *Ba813* [12, 19]. These data suggested that *Ba813* is no longer suitable as a specific chromosomal genetic marker for identification of *B*. *anthracis*, and *Ba813* (+) *B*. *cereus* is a major population among *B*. *cereus* which causes nosocomial BSI in Japan.

Recently, Akamatsu et. al. found a novel ST1420 harboring *Ba813* among nosocomial bacteremia cases in Japan [12]. However, the ST1420 strain was not included in this study. A combination of allele numbers of ST1420 showed the highest similarly to ST366 which differed in two of seven loci. In this study, ST366 strains were found in Group 2, but these isolates did not possess *Ba813*. Moreover, ST1050 strain (Group 1) possessing *Ba813* was a major ST strain isolated from patients with BSI in the hospital setting. However, ST366 and ST1050 strains were uncommon STs in previous isolates in Japan. A French multicenter study demonstrated that strains isolated from inpatients in nine hospitals had different M13-PCR fingerprints among hospitals, and the same strains in the hospitals were recovered from different patients and environments for up to a few years [29]. We observed that *Ba813* (+) *B*. *cereus* strains ST 365, ST167, and ST1050 were continuously isolated from patients with BSI for four years. Moreover, 29 different MLST genotypes from 53 isolates, including 16 novel STs, were identified. These data suggest that *B*. *cereus* is a ubiquitous opportunistic pathogen and can cause BSI in the hospital setting.

The lower prevalence of food poisoning-related gene (*hbl*) in *B*. *cereus* classified into Clade-1 including *Ba813*(+) strains agrees with the results of other studies [11, 18]. In contrast, most *B*. *cereus* strains isolated from feces in Japan were classified into Clade-2, and all four Clade-2 *B*. *cereus* (Group 4) had *hbl*. These data indicated that the virulence factors differ between *B*. *cerus* strains causing nosocomial BSI and those causing food poising. The virulence factors associated with non-gastrointestinal diseases are unknown, and other unknown virulence factors should be identified to improve the understanding of the pathogenicity of *B*. *cereus* during non-gastrointestinal nosocomial infection and whether *B*. *cereus* nosocomial infection varies depending on the host immune status.

Interestingly, *Ba813*(+) *B*. *cereus* and *Ba813* (-) *B*. *cereus* in Clade-1/Cereus-III were isolated from patients with significantly higher ages and higher Charlson co-morbidity indices compared to *B*. *cereus* Clade 2 which have *hbl*. A previous study revealed a difference in the detection rate of enterotoxins producing *B*. *cereus* strains isolates from blood culture between adults and newborns [29]. These data suggest that type of *B*. *cereus* strains associated with the onset of bacteremia might vary according to patient age.

Biofilms produced by *B*. *cereus* are considered as potential virulence factors and have been implicated in the development of medical device infection. Noteworthy, indwelling PVC rather than CVC was associated with catheter-related BSI caused by *B*. *cereus* [17, 30, 31]. However, the factors influencing biofilm production by pathogens including in *B*. *cereus* in PVC-related BSI are unknown. Previous studies showed that PVC-related BSI had a shorter duration from hospital admission compared to CVC-related BSI, and *Staphylococcus aureus* was detected more frequently in PVC-related BSI than in CVC-related BSI [32, 33]. We also previously found that methicillin-resistant *S*. *aureus* strains with high biofilm ability were isolated more frequently from patients with catheter-related BSI than from patients with other invasive infections and abscess formation [34]. In this study, *B*. *cereus* in Clade-1/Cereus-III including *Ba813*(+) strains (Group 1 and Group 2) showed significantly lower biofilm formation than *B*. *cereus strains* in Clade-1/non-Cereus-III and Clade-2 (Group 3 and Group4). Patients with BSI in Groups 1 and 2 had a significantly longer duration of bacteremia after admission and more frequent indwelling CVC compared to those with Groups 3 and 4 (Table 2). In addition, biofilm formation of *B*. *cereus* isolated from CVC-related BSI tended to be lower than that from PVC-related BSI (OD595 values: mean ± SD, 0.41 ± 0.19 *vs* 0.801 ± 0.7123, *p* = 0.058). These data suggest that *B*. *cereus* strains with high biofilm formation ability are associated with PVC-related BSI rather than CVC-related BSI in the hospital setting. It is still under discussion whether biofilm formation by pathogens is associated with poor clinical outcomes during bacteremia. However, for *B*. *cereus*, we found no association between biofilm production and poor clinical outcomes. *Bacillus cereus* strains with high biofilm formation (Group 3) ability were not associated with persistent bacteremia or mortality.

Patients with hematological malignancies and chemotherapy-induced neutropenia show a high risk for CNS complications caused by *B*. *cereus*. Previous studies suggested the gastrointestinal tract as the entry point of *B*. *cereus* involved in bacteremia and CNS complication among immunocompromised patients [1, 26, 35]. In our study, two of four neutropenic patients with hematological malignancies (50%) suffered from CNS complications during *B*. *cereus* bacteremia. These strains showed the same MLST profiles (ST-366) but did not possess *Ba813* or genes related to food poisoning. To und the relationships among *B*. *cereus* strains causing CNS complication, we analyzed MLST information and *B*. *cereus* strains obtained from blood culture samples of patients with hematological malignancy and CNS complications at other hospitals in Japan, as well previously published genome sequences of *B*. *cereus* strains that occurred with CNS complications [25] (S1 Fig). MLST analysis revealed that the pathogenic organisms clustered in two separate clades, suggesting that multiple distinct *B*. *cereus* strains regardless of whether they were genetically closely to *B*. *anthracis* or enterotoxin production cause CNS complications during bacteremia in patients with hematological malignancy.

Our study had some limitations. First, the sample size was small and the study was retrospectively performed in a single hospital; thus, our results may have been influenced by local clinical management practices and infection control policies. Indeed, the 30-day mortality rates among patients with *B*. *cereus* bacteremia in this study was much lower than those in previous reports [16, 36]. Infectious diseases specialists intervened in all cases to improve clinical outcome by administering appropriate antibiotics based on antibacterial susceptible testing and determining the duration of therapy. This may have affected our ability to compare clinical courses and outcomes. Second, we did not perform routine peripheral catheter tip culture to detect PVC-related BSI. However, infectious disease specialists carefully exclude the possibility of any other apparent sources of bacteremia. Third, we examined only anthracis-related and food poising-related virulence genes and biofilm production in *B*. *cereus*, and additional virulence factors of *B*. *cereus* reported should be analyzed to determine their association with the pathogenicity of nosocomial *B*. *cereus* BSI.

## Conclusions

*Ba813* (+) *B*. *cereus*, which is genetically closely related to *B*. *anthracis*, is isolated occasionally from the patients with elderly and indwelling CVC in nosocomial BSI in Japan. However, *Ba813* (+) *B*. *cereus* bacteremia is not associated with severity and high mortality in the hospital setting.

## Supporting information

S1 Table PCR primer sequences used in this study. (PDF)

S1 Fig Phylogenetic analysis based on MLST of *B*. *cereus* isolates from the patients with CNS complication. Differences of MLST among *B*. *cereus* isolates from the patients with CNS complications were compared. We collected three *B*. *cereus*, including this study, isolated from the patients with CNS complications at hospital A in Japan and four *B*. *cereus* isolates (Ref1-4) published by Rhee et. al [25]. *B*. *anthracis*, high pathogenic *B*. *cereus* (03BB102, 03BB108 and G9241) and *B*. *cereus* which were ATCC strains and three clinical isolates in Japan were selected from the MLST database (http://www.pubmlst.org/bcereus). A phylogenetic tree was drawn with MEGA X using the neighbor–joining methods [20]. Clade and lineage were defined as described previously [13]. Scale bar, 0.005 substitutions per nucleotide. Closed circle: *Ba813* (+) *B*. *cereus*; Ba: *B*. *anthracis*; Bc: *B*. *cereus*; ST; sequence type; CNS: central nerve systems.
(TIFF)
