# Supplementary material for: Ba813 harboring Bacillus cereus, genetically closely related to Bacillus anthracis, causing nosocomial bloodstream infection: Bacterial virulence factors and clinical outcome
Source: PLoS One. 2020 Jul 13;15(7):e0235771. doi: 10.1371/journal.pone.0235771 (PMC7357740; doi:10.1371/journal.pone.0235771)
Supplement: S1 Table — (PDF) [file pone.0235771.s001.pdf]

S1 Table: PCR primer sequences used in this study

Target gene	Primer sequence (5'→3')<br>(Upper: forward, Lower: reverse)	Reference
Ba813	TTAATTCACTTGCAACTGATGGG<br>AACGATAGCTCCTACATTTGGAG	[1]
hblA	ATTAATACAGGGGATGGAGAAACTT<br>TGATCCTAATACTTCTTCTAGACGCTT	[2]
hblC	CGAAAATTAGGTGCGCAATC<br>TAATATGCCTTGCGCAGTTG	[3]
hblD	AGGTCAACAGGCAACGATTC<br>CGAGAGTCCACCAACAACAG	[3]
cap	ACGTATGGTGTTTCAAGATTCATG<br>ATTTTCGTCTCATTCTACCTCACC	[4]
nheA	GTTAGGATCACAATCACCGC<br>ACGAATGTAATTTGAGTCGC	[5]
nheB	TTTAGTAGTGGATCTGTACGC<br>TTAATGTTCGTTAATCCTGC	[5]
nheC	TGGATTCCAAGATGTAACG<br>ATTACGACTTCTGCTTGTGC	[6]
pag	CAGAATCAAGTTCCCAGGGG<br>TCGGATAAGCTGCCACAAGG	[6]
lef	CTTTTGCATATTATATCGAGC<br>GAATCACGAATATCAATTTGTAGC	[6]
cya	GGTTTAGTACCAGAACATGC<br>CGGCTTCAAGACCCC	[6]
cytK	AACAGATATCGGTCAAAATGC<br>CGTGCATCTGTTTCATGAGG	[7]
glpF	GCGTTTGTGCTGGTGTAAGT<br>CTGCAATCGGAAGGAAGAAG	[8]
gmk	ATTTAAGTGAGGAAGGGTAGG<br>GCAATGTTCACCAACCACAA	[8]
ilvD	CGGGGCAAACATTAAGAGAA<br>GGTTCTGGTCGTTTCCATTC	[8]
pta	GCAGAGCGTTTAGCAAAAGAA<br>TGCAATGCGAGTTGCTTCTA	[8]
pur	CTGCTGCGAAAAATCACAAA<br>CTCACGATTCGCTGCAATAA	[8]

pycA	GCGTTAGGTGGAAACGAAAG<br>CGCGTCCAAGTTTATGGAAT	[8]
tpi	GCCCAGTAGCACTTAGCGAC<br>CCGAAACCGTCAAGAATGAT	[8]

Ba813 (a 277-bp long chromosomal DNA fragment from B. anthracis), B. anthracis-related virulence factor: *pag*, *lef*, *cya* (protective antigen, lethal factor and edema factor; encoded at the pXO1 in B. anthracis) and *cap* (capslur antigen; encoded at the pXO2 in B. anthracis), food poising-related virulence factor; *hblA*, *hblC*, *hblD* (hemolysin BL), *nheA*, *nheB*, *nheC* (non-hemolytic toxin) and *cytK* (cytotoxinK).

Reference

[1] G. Patra, P. Sylvestre, V. Ramisse, J. Therasse, J.L. Guesdon, Isolation of a specific chromosomic DNA sequence of *Bacillus anthracis* and its possible use in diagnosis. FEMS Immunol Med Microbiol 1996; 15:223-231.

[2] E. Wehrle, M. Moravek, R. Dietrich, C. Burk, A. Didier, E. Martlbauer, Comparison of multiplex PCR, enzyme immunoassay and cell culture methods for the detection of enterotoxinogenic *Bacillus cereus*. J Microbiol Methods 2009; 78:265-270.

[3] M. Moravek, M. Wegscheider, A. Schulz, R. Dietrich, C. Burk, E. Martlbauer, Colony immunoblot assay for the detection of hemolysin BL enterotoxin producing *Bacillus cereus*. FEMS Microbiol Lett 2004; 238:107-113.

[4] H. Ellerbrok, H. Nattermann, M. Ozel, L. Beutin, B. Appel, G. Pauli, Rapid and sensitive identification of pathogenic and apathogenic *Bacillus anthracis* by real-time PCR. FEMS Microbiol Lett 2002; 214:51-59.

[5] M.H. Guinebretiere, V. Broussolle, C. Nguyen-The, Enterotoxigenic profiles of food-poisoning and food-borne *Bacillus cereus* strains. J Clin Microbiol 2002; 40:3053-3056.

[6] V. Ramisse, G. Patra, H. Garrigue, J.L. Guesdon, M. Mock, Identification and characterization of *Bacillus anthracis* by multiplex PCR analysis of sequences on plasmids pXO1 and pXO2 and chromosomal DNA. FEMS Microbiol Lett 1996; 145:9-16.

[7] E. Ghelardi, F. Celandroni, S. Salvetti, C. Barsotti, A. Baggiani, S. Senesi, Identification and characterization of toxigenic *Bacillus cereus* isolates responsible for two food-poisoning outbreaks. FEMS Microbiol Lett 2002; 208:129-134.

[8] F.G. Priest, M. Barker, L.W. Baillie, E.C. Holmes, M.C. Maiden, Population structure and evolution of the *Bacillus cereus* group. J Bacteriol 2004; 186:7959-7970.
